# Performance and acceptability of the Stressful Life Events Screening Questionnaire in a chronic pain population: a mixed-methods study

**DOI:** 10.1097/PR9.0000000000001072

**Published:** 2023-04-24

**Authors:** Lene Therese Bergerud Linnemørken, Helle Stangeland, Silje Endresen Reme, Synne Øien Stensland

**Affiliations:** a Division of Emergencies and Critical Care, Department of Research and Development, Oslo University Hospital, Oslo, Norway; b Faculty of Social Sciences, Department of Psychology, University of Oslo, Oslo, Norway; c Division for Health Services, Department of Health Services Research, Norwegian Institute of Public Health, Oslo, Norway; d Norwegian Centre for Violence and Traumatic Stress Studies, Oslo, Norway; e Division of Clinical Neuroscience, Department of Research and Development, Oslo University Hospital, Oslo, Norway; f Faculty of Medicine, Institute of Clinical Medicine, University of Oslo, Oslo, Norway; g Division of Emergencies and Critical Care, Department of Pain Management and Research, Oslo University Hospital, Oslo, Norway

**Keywords:** The Stressful Life Events Screening Questionnaire, Chronic pain, Posttraumatic stress disorder, Comorbidity, Trauma screening

## Abstract

**Introduction:**

Pain-related fear, anxiety, and avoidance may play key roles in the chronification of pain and related disability. For practitioners, knowledge about the source or drivers of these fears, including patients' exposure to potentially traumatic events (PTEs) and related posttraumatic stress symptoms, could be particularly helpful in guiding their treatment approach.

**Objectives:**

We aimed to investigate whether the use of a brief screening for PTEs could help inform chronic pain treatment.

**Methods:**

The performance and acceptability of the Stressful Life Events Screening Questionnaire (SLESQ) was assessed among 567 adult patients (59% women, mean age 48.1 years) meeting at a hospital outpatient pain clinic. The sensitivity, specificity, and 20 months temporal stability of the SLESQ, assessing exposure to 14 specific trauma types followed by a 15th item capturing exposure to “other events,” were assessed through digital administration and follow-up interviews with 55 participants. The qualitative responses of 158 participants reporting exposure to “other events” were reviewed and assessed based on fulfillment of the A Criterion for traumatic events in the Diagnostic and Statistical Manual of Mental Disorders, Fifth Edition. The acceptability of the SLESQ was assessed in clinical interviews with 12 participants.

**Results:**

The SLESQ demonstrated acceptable sensitivity (70.0%), high specificity (94.9%), and moderate temporal stability (κ = 0.66, *P* < 0.001). Participants' qualitative elaborations of “other events” were largely (76.3%) consistent with Criterion A events. The screening was well accepted and welcomed.

**Conclusion:**

The results indicate that the use of a brief screening for potential trauma may be helpful to guide clinical practice in chronic pain settings.

## Abstract 2

Supplemental Digital Content is Available in the Text.

The Stressful Life Events Screening Questionnaire demonstrates satisfactory sensitivity, specificity, temporal stability, and acceptability in a chronic pain clinic and can help guide clinical practice within these settings.

## 1. Introduction

Pain-related fear, anxiety, and avoidance play key roles in chronification of pain and related disability.^23,26,41^ In clinical practice, knowledge of potential factors underlying or driving these fears could be particularly helpful for providing need-based, patient-centered care and increase treatment effectiveness.^20,29^ As chronic pain is merely a symptom-based diagnosis, choice of treatment and related outcome largely rely on practitioners' assessment and understanding of potential underlying causal or mediating agents of the complaints and comorbidity in question. More than one in 5 patients meeting at pain clinics report high levels of posttraumatic stress symptoms, including reexperience, avoidance, negative alterations in cognitions, and mood or physiological arousal, following exposure to potentially traumatic events (PTEs).^1,22^ Yet, systematic screening of chronic pain patients' exposure to injuries, medical incidents, and other PTEs is often lacking.^31^ Presence of posttraumatic stress disorder (PTSD) in chronic pain patients is associated with higher levels of anxiety and pain catastrophizing^18,22,37^ and adversely impact prognosis.^6,9,10^ Chronic pain patients with these symptoms may need tailored interventions directed at their fear-related beliefs,^12^ and a lack of systematic screening of chronic pain patients' exposure to PTEs may, therefore, hinder best practice.^31^

A pertinent question is whether the use of a brief screening for PTEs could be helpful to capture the range of trauma exposure experienced by chronic pain patients in hospital clinics. Yet, the acceptability and performance of standardized screening questionnaires for PTEs in chronic pain populations remain to be evaluated. Existing questionnaires, such as the commonly used Stressful Life Events Screening Questionnaire (SLESQ),^14^ have largely been developed for screening of PTE exposure in general, nontreatment seeking populations, and validated among college students,^14^ veterans,^15^ and victims of assault.^42^ However, in contrast to these populations, chronic pain patients referred to outpatient hospital pain clinics make up a highly selected treatment-seeking population, commonly experiencing considerable functional impairment. Given the phenomenological, conceptual, and observed partial overlap between experiences of PTEs, pain, and posttraumatic stress symptoms,^6,36^ there is, therefore, a chance that chronic pain patients' perception, accept, response to, and need of PTEs' screenings could deviate from that of the general population.

In particular, little is known about such questionnaires' ability to identify events experienced as traumatic by chronic pain patients. For example, when screened for trauma exposure, a third (34%) of chronic pain patients in a hospital outpatient pain clinic recently reported exposure to “other [potentially traumatic] events.”^22^ This category targets events not listed among the standardized screening questions, encompassing exposure to severe violence, accidents, disaster and other life-threatening events.^22^ Positive responses to this “other” category are currently considered to fulfill the A Criterion for PTSD (trauma exposure) in the Diagnostic and Statistical Manual of Mental Disorders, Fifth Edition (DSM-5),^3^ although little is known about the actual experiences patients refer to. To increase understanding of patients' experiences, inform diagnostics, and help facilitate more effective, need-based, treatment processes, it is imperative to improve assessment procedures and expand current knowledge of chronic pain patients' potentially traumatic experiences. Without such knowledge, symptoms of psychological disorders related to trauma exposure may be misinterpreted as expression of patients' pain conditions or they may be overlooked completely and result in lowering the chance of treatment success.

This study evaluates the performance and acceptability of one of the most commonly used instruments for the assessment of potential trauma exposure, the SLESQ,^14^ applied in a chronic pain clinic. The aims of this study were threefold: (1) investigate the sensitivity, specificity, and temporal stability of the SLESQ when applied in a hospital outpatient pain clinic; (2) examine the proportion of reported “other events” fulfilling criteria as potentially traumatic (Criterion A); and (3) investigate the acceptability of a screening for PTEs among patients meeting for assessment in a hospital outpatient pain clinic.

## 2. Methods

### 2.1. Participants

**Figure 1.**
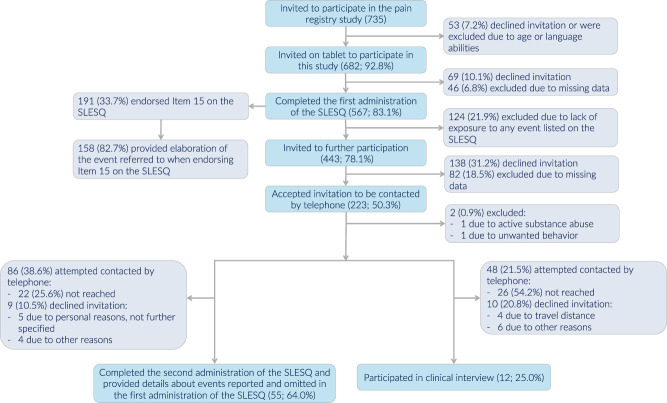
Flowchart showing the inclusion process. SLESQ, Stressful Life Events Screening Questionnaire.

All adult patients (defined as patients turning 18 in the year of data collection) meeting for pain assessment at the largest hospital outpatient pain clinic in Norway from March to December 2019 were consecutively recruited. The clinic provides interdisciplinary assessment and treatment to patients with chronic pain^40^ from all parts of Norway. Exclusion criteria were limited Norwegian abilities and insufficient data (the half rule was applied for scale measures). Two subsamples were consecutively selected for participation in telephone interviews (n = 55) and clinical interviews (n = 12) (Fig. 1).

### 2.2. Procedure

Participants completed web-based questionnaires providing data on sociodemographics, clinical characteristics, and exposure to PTEs in the clinic's waiting room before their appointment. Based on reports of exposure to PTEs, participants who reported exposure to nonspecified, “other events” were asked to elaborate on the event in an open text field. Participants who reported exposure to at least one PTE were invited to participate in the following:

(1) A telephone interview targeting the performance of the SLESQ, conducted by trained psychology students. Recruitment ended when saturation was reached (n = 55, June 2021). The average time interval between the 2 administrations of the SLESQ was 20 months (range: 14–24).
(2) A clinical interview targeting their experience with the screening for PTEs at the pain clinic conducted by a trained clinical psychologist (L.T.B.L.), face-to-face at the clinic (n = 9) or over telephone (due to COVID-19 restrictions, n = 3). Recruitment ended when saturation was reached (n = 12, December 2020).

### 2.3. Measures

To assess lifetime exposure to PTEs in line with the diagnostic Criterion A for PTSD in the DSM-5,^3^ a modified, Norwegian translation of the SLESQ—Revised^16,39^ (Table 1) was applied. The self-report questionnaire has demonstrated satisfactory psychometric properties in diverse populations,^2,14,16^ but it has yet to be examined in chronic pain populations. Participants were asked to indicate exposure to the following 15 event categories by selecting “Yes” or “No”: (1) life-threatening illness, (2) life-threatening accident, (3) natural disaster, (4) robbery or assault with use of physical force or weapon, (5) unnatural death of a very close person, (6) physically forced or threatened to sexual activity, (7) forced to touch others' or others touching one's own private parts, (8) violence as a child, (9) violence as an adult, (10) humiliation by a family member, (11) humiliation by someone outside the family, (12) threatened with a weapon, (13) witnessed life-threatening event, (14) other life-threatening situation involving own serious illness or life-threat, and (15) other extremely frightening or horrifying situation or one in which one felt extremely helpless.

**Table 1:** Temporal stability of all items of the modified Stressful Life Events Screening Questionnaire—revised.

The Stressful Life Events Screening Questionnaire—revised (modified)	Temporal stability (κ)
1. Have you ever had a life-threatening illness?	0.67*
2. Were you ever in a life-threatening accident?	0.66*
3. Have you ever been affected by a natural disaster?	1.0*
4. Was physical force or a weapon ever used against you in a robbery or mugging?	0.64*
5. Has an immediate family member, romantic partner, or very close friend died because of accident, homicide, or suicide?	0.74*
6. At any time, has someone (parents, other family member, partner, acquaintance, or others) by use of physical force or threats forced you to having intercourse or oral sex or anal sex against your own will?	0.66*
7. Other than experiences mentioned in earlier questions, has anyone ever touched private parts of your body or made you touch their body against your wishes?	0.70*
8. When you were a child, did a parent, caregiver, or other person ever slap you repeatedly, beat you, or otherwise attack or harm you?	0.65*
9. As an adult, have you ever been kicked, beaten, slapped around, or otherwise physically harmed by a romantic partner, date, family member, acquaintance, or someone else?	0.74*
10. Has a parent, romantic partner, or family member repeatedly ridiculed you, put you down, ignored you, or told you were no good?	0.63*
11. Has someone outside the family, such as classmates or colleagues, repeatedly ridiculed you, put you down, ignored you, or told you were no good?	0.80*
12. Other than the experiences already covered, has anyone ever threatened you with a weapon such as a knife or gun?	0.54*
13. Have you ever been present when another person was killed, seriously injured, sexually or physically assaulted?	0.46*
14. Have you ever been in any other situation where you were seriously injured or your life was in danger (eg, living in a war zone or terrorist attack)?	0.64*
15. Have you ever been in any other situation that was extremely frightening or horrifying, or one in which you felt extremely helpless, that you haven't reported?	0.17†

* Significance level *P* < 0.001.

† Significance not reached.

For the clinical interviews, a semi-structured interview guide developed for this study was applied. The interview consisted of 14 open-ended questions, of which 5 are analyzed here (for the complete interview guide, see Suppl. 1, available at http://links.lww.com/PR9/A187). Participants were asked to reflect on their experiences with the screening for PTEs using the SLESQ at the clinic, whether listed events were appropriate, and if trauma-related thoughts were triggered. Furthermore, they were asked to share their opinions on making the screening standard procedure at the clinic and report any concerns regarding whether an increased focus on trauma could disturb the pain treatment.

### 2.4. Data analysis

SPSS (version 27) was used for quantitative analyses and NVivo (version 12) for qualitative analyses. Descriptive statistics were used to analyze demographic and clinical characteristics. One-way analysis of variances and Chi-squared tests were conducted to analyze group differences on demographical data between the total sample and the subsamples.

The sensitivity (ie, capacity to identify Criterion A events) and specificity (ie, capacity to exclude non-Criterion A events) of the SLESQ were assessed with a subsample of participants (n = 55; Fig. 1). Events reported by participants in the first administration of the SLESQ were classified as Criterion A or non-Criterion A events according to the criteria for PTSD in DSM-5,^3^ based on participants' verbal descriptions of these events. Events omitted in the second administration were registered and classified in line with earlier reported events.

The temporal stability as well as the test–retest reliability were assessed by the use of Cohen kappa statistics.

The proportion of Criterion A events reported on item 15 (“other event”) was investigated by classifying qualitative responses to this item from a subsample of participants (n = 158) in the first administration as Criterion A or non-Criterion A events. Classifications were performed according to a predefined coding manual developed for this study, that aimed to outline conservative criteria for Criterion A events (Suppl. 2, available at http://links.lww.com/PR9/A187). Classifications were made blindly, and Cohen kappa statistics was applied to determine interrater reliability.^28^

The acceptability of the SLESQ was assessed using systematic text condensation (STC),^24,25^ a useful tool for qualitative analyses in chronic pain research that allows for analyses of qualitative data from a descriptive and explorative approach.^33–35^ Systematic text condensation allows for analyses of qualitative data from a descriptive and explorative approach and is performed in 4 steps: (1) gain an overall impression of the data and identify main themes, (2) identify meaningful units and sort these, (3) split groups of meaningful units into smaller units and transform these to a more general format (condensation), and (4) summarize the data and present the prominent themes based on the condensates from the previous step. The audio files were transcribed, and 2 investigators (L.T.B.L. and S.E.R.) examined the transcripts and identified reoccurring and prominent themes (step 1). One investigator (L.T.B.L.) performed the following steps (step 2–4) under close guidance of a senior researcher (S.E.R.), and overall consensus was reached without complications for all main themes.

### 2.5. Ethical aspects

The Regional Committees for Medical and Health Research referred the project to the Data Protection Officer at Oslo University Hospital, where it was approved (ref. 18/25982). The Helsinki Declaration was followed, and written informed consents were provided by all participants. Patients' right to withdraw from participation without any consequences for treatment at the clinic was highlighted. An experienced clinical psychologist was available for support at all times during participation. Participants in clinical interviews each received a small gift card as compensation for travelling expenses.

## 3. Results

### 3.1. Sample characteristics

In total, 567 chronic pain patients participated (59.4% females, mean age = 48.1 years; SD = 15.6, range = 17–92), of whom the majority were unemployed (283 of 459; 61.7%) and most lived alone (331 of 466; 71.0%). The mean pain duration was 7.2 years (SD = 7.6, range = 0–45), and the average pain intensity was 7.2 (SD = 1.8, range = 2–10) on the Numerical Rating Scale (NRS)^8^ ranging from 0 (“not at all”) to 10 (“worst imaginable”). The majority (n = 443, 78.1%) reported exposure to at least one PTE. In total, 1440 events were reported (mean = 3.3). “Other event” (n = 191; 33.7%) and “life-threatening disease” (n = 160; 28.2%) were most frequently reported. Additional sample characteristics are described elsewhere.^22^ The 55 participants in the telephone interview and the 12 participants in the clinical interviews were largely comparable with the total sample population for selected sociodemographics and pain diagnoses (Suppl. 3, available at http://links.lww.com/PR9/A187).

### 3.2. Sensitivity, specificity, and temporal stability

In the follow-up telephone interview of 55 participants about 20 months after the first administration, conducted to examine the performance of the SLESQ, 196 (87.5%) of 224 reports of exposure to PTEs fulfilled Criterion A (true positives). Over the 20-month period, the 55 participants added a total of 96 events and omitted 27 in the second administration of the SLESQ compared with the first. Among the 96 added events (mean = 2.5, range: 1–6), 85 (88.5%) fulfilled the A Criterion, classifying as false negatives in the first administration of the SLESQ. The sensitivity was 70.0%. Four participants (7.3%) reported refraining from reporting events in the first administration, stating that they did not feel comfortable with providing sensitive information on the tablet or they were not ready to report their experiences.

The 55 participants in the first administration of the SLESQ reported not being exposed to a total of 601 event categories. Among these responses, 516 were classified as true lack of exposure or non-Criterion A events (true negatives) and 28 responses were classified as actual exposure to Criterion A events (false negatives). The specificity was 94.9%.

The temporal stability of the SLESQ over a 20-month time period was moderate (κ = 0.66, *P* < 0.001). The correlations varied between items (Table 1), and the median kappa was 0.65.

### 3.3. Proportion of Criterion A events reported on Item 15 of the Stressful Life Events Screening Questionnaire

Among the third of the participants (191 of 567; 33.7%) who reported exposure to nonspecified “other PTEs” in the SLESQ, 158 (82.7%) provided written descriptions. The 158 answers were classified as 224 separate types of events (mean = 1.4, range: 1–5), of which 110 were excluded from further analyzes due to insufficient information (eg, responding “Fire”). The interrater agreement for the classification of the remaining responses as Criterion A or non-Criterion A events was moderate (κ = 0.68, *P* < 0.001). Among the 114 analyzed “other events”, 87 (76.3%) reached the predefined criteria for Criterion A events, whereof 39 (44.8%) qualified as PTEs predefined in the SLESQ (Table 1), whereas 48 (55.2%) were not covered by the predefined categories. These 48 “other PTEs” not listed elsewhere in the SLESQ were categorized in 2 subgroups as (1) *exposure to serious or life-threatening illness in a close person* (n = 23; 47.9%) or (2) *miscellaneous events* (n = 25; 52.1%). The events most frequently reported in the latter category were (1) *closeness to a serious event* (eg, working with injured individuals, n = 5; 20.0%), (2) *attempted rape or assault* (n = 3, 12.0%), and (3) *witnessing a serious event happen to another person* (n = 3; 12%). Among the 27 (23.7%) “other events” not fulfilling Criterion A, *chronic pain* (n = 9, 33.3%) and *major life events* (eg, death of a grandparent, n = 7, 25.9%) were most frequently reported.

### 3.4. Acceptability

From the qualitative interviews with 12 participants, 4 main themes were identified. Original quotes are translated and used to illustrate and exemplify.

(1) Overall experience

All participants reported that they considered questions about potential trauma exposure useful and relevant within the setting. One participant reported: “When I have bad periods in life, I have more pain and I let it affect me to a much larger degree. To me, it is obvious that the 2 [bad periods and pain severity] are connected.” Three participants described the screening as a positive experience, reporting that it was “health promoting” or “good” to receive the questionnaire, but one added that it also felt “a little painful” as it evoked unpleasant memories. A majority of the participants reported the screening to be a neutral experience; 6 participants described the screening as “nonproblematic” or “okay.” Two participants reported finding it difficult to complete the screening because of their professional background and language difficulties.

(2) Individual consequences

Expressions of gratitude for targeting potential trauma exposure at the pain clinic were spontaneously given by participants; “It was like, yes! Finally, someone is asking me this! That's how I felt. Because no one asks. If it's doctors, my general practitioner, others I'm around… It [the experienced event] is just mentioned in passing, and then we move on.” Eight participants reported no increase in trauma-related thoughts after the screening, and 4 participants reported an increase in such thoughts for a maximum period of “some days.” Only one participant reported the increase in thoughts to be bothersome, but they later added that reflecting on the experiences made them easier to handle. Two of the participants who reported increased, but not bothersome, thoughts reported that the screening either to some degree made the event they had experienced more real or activated a thought process experienced as explanatory. None of the participants reported inability to handle the thoughts or a need to seek further medical care after the screening, and none contacted the dedicated health personnel available for support during or within 6 months after participation.

(3) Reflections on screening for PTEs as a part of the pain assessment

Most participants expressed a positive attitude towards including screening for PTEs in the standardized pain assessment at the clinic, reporting no concerns about the screening interfering with pain assessment or treatment. One-third of participants reported thinking it would be beneficial, highlighting the need for increased knowledge about connections between physical and psychological aspects of chronic pain and that a holistic treatment approach requires addressing psychological aspects. One participant reported: “The advantage [of performing trauma screenings at the clinic] is that you see all of me, and not just what I'm coming [to the clinic] for.” Two participants supported an integrated screening for potential trauma if certain conditions were met (eg, sufficient background information should be provided). Only 2 participants expressed worries about the screening disrupting the pain treatment, by activating unpleasant memories or take focus away from the physical aspects of the pain condition. Concerns regarding the clinics' ability to follow-up on the data provided and concerns about other patients' reactions were also reported.

(4) Perception of the SLESQ

Most participants reported that they found the events listed in the SLESQ to be relevant and appropriate. However, one participant emphasized the need for questionnaires customized for first responders. They reported: “I felt a bit uneasy, to be honest. I realized this [questionnaire] was not designed for people like me.” Other participants suggested adding medical procedures, difficult living situations, and chronic pain to the list of PTEs. Some uncertainty about the definitions of words used (“ridiculed” in Item 10 and “assault” and “sexual assault” in Item 4 and 13) were also expressed, and one participant reported difficulties completing the SLESQ (eg, options did not correspond to their experiences).

## 4. Discussion

This study investigated the performance and acceptability of potential trauma screening in a chronic pain clinic population. The results add support for the use of the SLESQ as a screening tool for PTEs in this setting. The SLESQ performed well for sensitivity, specificity, and temporal stability and a high proportion of the reported exposure to “other PTEs” qualified as Criterion A events. Results also indicated that in addition to the PTEs specifically screened for, patients with chronic pain commonly report exposure to several other PTEs not listed on the SLESQ. Furthermore, qualitative interviews supported the acceptability of screening for potential trauma in a pain clinic setting.

The SLESQ's ability to detect Criterion A events among chronic pain patients was lower than expected, but still acceptable (70%), and in line with results from the original validation with college students.^14^ In our study, 89% of the events participants omitted from the first administration of the SLESQ classified as Criterion A events, whereas 37% of the omitted events qualified in the original study. The higher number in this study thereby contributed to a lower sensitivity of the SLESQ in this chronic pain population. As further discussed below, inconsistencies in the number of reported events between the first and second administration of the SLESQ may be due to a range of methodological factors. Overall, the sensitivity found suggests that the SLESQ can be applied with chronic pain patients, while also keeping in mind the risk of misclassifying patients.

The SLESQ's ability to dismiss the lack of exposure or subthreshold events was very high (95%). This may be a result of the predefined criteria for which events should be classified as Criterion A events. In line with the criterion applied by the authors of the SLESQ, a conservative criterion was used to minimize the risk of subthreshold events being misclassified. This strict criterion did, however, also increase the possibility of misclassifying individuals exposed to potential trauma as not exposed. As such, a less conservative interpretation of the DSM-5 criterion for PTSD^3^ might be more appropriate in clinical settings.

The SLESQ demonstrated moderate temporal stability over 20 months, slightly lower than results from the original study.^14^ Inconsistent reporting may not be due to the longer time interval applied between the 2 administrations of the SLESQ, as the temporal stability corresponds with the findings in the original article (20 months vs 2 weeks).^14^ As more than one-third of individuals exposed to PTEs find it challenging to answer “yes” or “no” to questions about such exposure, this may account for some of the inconsistent reporting.^38^ Also, as it is not uncommon that participants report an increased number of events in later potential trauma screenings,^11,21^ there is a chance that the first administration affects later administrations (eg, by activating memories^11^). Furthermore, as 7% of the participants intentionally refrained from reporting events when they completed the web-based SLESQ on a tablet, it is plausible that talking to an empathic, trained person may increase participants' sense of security and trust and thereby lower the burden of disclosing adverse events during the second in-person assessment.^14,19^ This indicates the utility of integrating questions about PTEs in clinical consultations.

The test–retest challenges with Item 15 (reports of “other events”) may be explained by the lack of specification of the item, but the wording may also have influenced the responses. The SLESQ's authors initially considered 2 open event categories: “other life threat” and “other horrifying event.”^14^ Although the former demonstrated a strong 2 weeks stability in a screen–telephone interview condition, such stability was absent for the latter. In the version of the SLESQ applied in this study, participants were asked to report exposure to events experienced as “*extremely frightening or horrifying or one in which you felt extremely helpless*.” The exclusion of the words “life threat” may, therefore, have weakened the temporal stability for this item.

More than 3 out of 4 events reported in the “other event” category qualified as Criterion A events. However, a substantial number of events reported on this item were not listed earlier in the questionnaire. It is possible that exposure to these events and related symptoms of PTSD may fuel chronic pain patients' frequency and level of complaints, similarly to the PTEs listed in the SLESQ. Furthermore, almost half of the reported Criterion A events not listed described a close person's disease. Although other screening questionnaires for potential trauma such as the Life Events Checklist^43^ have included reporting of witness experiences for all events listed, the SLESQ only requests this on certain items. The lack of targeting of these events may be considered a weakness of the SLESQ, but as responding positively to Item 15 qualifies as potential trauma exposure, the questionnaire's aim is still reached. Hence, the results suggest that inclusion of an “other event” category provides a flexibility that may compensate for the need of a more specifically adjusted PTEs screening questionnaire for identifying such exposure in chronic pain populations.

The clinical interviews showed that screenings for PTEs are well accepted and welcomed by patients in a pain clinic setting. All participants reported the overall experience of the screening to be a positive or neutral experience. In particular, participants reported that the screening was experienced as helpful. A large majority reported that the screening made it possible to finally address the interplay between their pain condition and the traumatic event(s) in a clinical setting. A desire to be met with a holistic treatment approach to their chronic pain condition was expressed by a majority of the participants, and the screening for PTEs was considered necessary to accomplish this.

Strategies have been developed to ensure that exposure to potential trauma is assessed in clinical settings, and trauma-informed care has gained increased attention as a beneficial approach to trauma-exposed individuals in healthcare services.^17,32^ Still, physicians often avoid screening their patients for potential trauma exposure due to concerns about causing the patient discomfort.^4,27,44^ Our results indicate that such concerns are not a valid argument for avoiding these screenings as most participants expressed positive attitudes towards such screenings in this context. This is also in line with earlier findings suggesting that most patients are comfortable with being asked by health professionals about adverse events.^7,13,38^ The results of this study indicate that patients are not only willing to complete such assessments but welcome them.

The participants also mentioned a few concerns regarding the screening for exposure to PTEs. The issue most frequently reported was uncertainty regarding the clinics' ability to follow-up on the information they provided. This feedback from patients speaks to a broader, professional ethical obligation in health care, emphasizing the necessity of follow-up after the identification of risk in patients. Few chronic pain clinics are currently specialized in treatment of trauma-related conditions, and thus, many patients with such symptoms are commonly referred to other healthcare services. Nevertheless, the high level of comorbidity and interrelated causal mechanisms of chronic pain and PTSD indicate that an integrated treatment approach in pain clinics may be preferable.^5,30^

This study has some limitations. First, establishing clear criterions for which events that should qualify as Criterion A events was challenging, particularly for events with variable severity. Second, the results could not be compared with the performance of similar screening questionnaires for PTEs in chronic pain populations, as none have so far been validated for this use. Third, the generalizability may be limited based on the characteristics of the study population.

In conclusion, the results of this study suggest that a brief questionnaire screening for PTEs can be helpful to screen for such exposure in chronic pain populations, and thereby guide practice in chronic pain settings. A brief screening should, however, be followed by more comprehensive assessments of reported potential trauma exposure, severely adverse life events, and related symptomatology to best inform the diagnostic process. The results further suggest that screening for potential trauma exposure is well-accepted and welcomed by patients at a hospital outpatient pain clinic.

## Disclosures

The authors declare that they have no conflict of interest related to the research or to the manuscript. No funding was received for this work.

## Appendix A. Supplemental digital content

Supplemental digital content associated with this article can be found online at http://links.lww.com/PR9/A187.

## Supplementary Material
