# Supplementary material for: Performance and acceptability of the Stressful Life Events Screening Questionnaire in a chronic pain population: a mixed-methods study
Source: Pain Rep. 2023 Apr 24;8(3):e1072. doi: 10.1097/PR9.0000000000001072 (PMC10129107; doi:10.1097/PR9.0000000000001072)
Supplement: Supplementary file 1 [file painreports-8-e1072-s001.pdf]

Supplementary File 1

Interview Guide

Individual interviews with patients in the study “Chronic pain and psychological trauma”.

1. How long has it been since you experienced the event?

2. Have you experienced more than one serious event during your lifetime?
    a. If yes, please describe

3. How did you feel about completing a questionnaire about serious life events here at the pain clinic?
    a. Was anything odd or strange?
    b. Was it useful in any way?

4. Do you think the events listed were appropriate?
    a. If no, what do you think should have been different?

5. Have you been thinking more about the event(s) after you completed the questionnaire?
    a. If yes, has it been bothersome to you?
    b. If yes, do you know who to contact to get help managing these reactions?

6. How do you feel about making this a standard routine at the pain clinic - that is, ask all patients meeting for their first appointment to complete a questionnaire about serious life events?
    a. Can you think of any advantages by doing this?
    b. Can you think of any disadvantages related to doing this?

7. Are you worried that increased focus on serious life events may interfere with the pain treatment in any way?

8. Do you think that there is a connection between what happened and that you have chronic pain/that what happened has affected the pain anyhow (depending on which condition came first)?

9. Do you think the event affects your pain today?

10. Do you think the event affects your function today?

11. Do you think the event affects your quality of life today?

12. Do you consider the help you have received to manage this event(s) to be good, if it has been necessary?
    a. If yes, what was it that helped you?
    b. If no, what do you think might could have helped?

13. What do you think about offering patients who meet for pain assessment and treatment a psychological treatment targeting both the pain condition and the consequences of the event?
    a. Does it seem like a useful treatment option?
    b. Or does it seem unnecessary or burdening in any way?

14. Is there something you would like to add – that I have not asked you?

Supplementary File 2

Coding Manual

Coding manual for open responses to item 15 on the Stressful Life Events Screening Questionnaire (SLESQ) [1]: Have you ever been in any **other** situation that was extremely frightening or horrifying, or one in which you felt extremely helpless, that you haven't reported?

1. **Event is listed earlier**
   This category includes all cases in which the participant describes an event that is listed in the 14 preceding items of SLESQ. This includes:
   - Life-threatening diseases
   - Life-threatening accidents
   - Natural disasters
   - Robbery or mugging with the use of physical force or a weapon
   - Someone close dying by accident, homicide, or suicide
   - Being physically forced or threatened to intercourse, oral sex, or anal sex
   - Being touched on private parts against own will, or being forced to touch another person's private parts
   - Being kicked, beaten, or otherwise attacked or harmed by an adult as a child
   - Being kicked, beaten, slapped or otherwise physically harmed as an adult
   - Being repeatedly ridiculed, humiliated, or told one is not good enough by a partner or a family member
   - Being repeatedly ridiculed, humiliated, or told one is not good enough by someone outside the family
   - Being threatened with a weapon
   - Being present when another person was killed, seriously injured, or sexually or physically assaulted
   - Other situations where one was seriously injured or one's life was in danger, such as war zones or terrorist attacks

   To be included in this category, the participant must have experienced, been present at, or been directly affected by the event.

2. **Event qualifies as Criterion A but is not listed**
   This category includes all cases in which the participant describes an event that is not listed on SLESQ, but still qualifies as Criterion A for posttraumatic stress disorder (PTSD) in the fifth version of the Diagnostic and Statistical Manual of Mental Disorders (DSM-5) [2]. According to DSM-5, the event must include actual or threatened death, serious injury, or sexual violence. All events placed in this category should be further classified as a 2a, 2b, or 2c event.

   a. **Participant describes serious or life-threatening illness in person encountered while working as first responder**
      This category includes all cases in which the participant has witnessed serious or life-threatening illness in a person while working as a first responder (e.g., law enforcement officer, paramedic, emergency medical technician, and firefighter). The event should be included in this category only if the participant was on the

site of the event, he or she was directly affected by the event, and the event involved a threat to health and life (e.g., paramedics arriving at the site of someone's life-threatening asthma attack or ruptured main artery).

b. **Participant describes close person's serious or life-threatening illness**
This category includes all cases in which the participant describes an event involving serious or life-threatening illness in a person close to them. If the participant work as a first responder but was off duty when the event happened to someone close to him or her, the event should be placed in this category.

c. **Participant describes event not related to another person's illness**
This category includes all cases in which the participant describes an event not involving serious or life-threatening illness in another person (2a or 2b), but the event still qualifies as Criterion A for PTSD in DSM-5.

3. **Event does not qualify as Criterion A**
This category includes all cases in which the participant describes an event that does not qualify as Criterion A for PTSD in DSM-5. This might be an event that the participant considers to be traumatic but has not involved actual or threatened death, serious injury, or sexual violence. All events placed in this category should be further classified as 3a, 3b, or 3c events.

a. **Participant describes chronic pain**
This category includes all cases in which the participant describes chronic pain, and no further information regarding the involvement of actual or threatened death, serious injury, or sexual violence is provided by the participant.

b. **Participant describes anxiety**
This category includes all cases in which the participant describes anxiety symptoms or disorders, and no further information regarding involvement of actual or threatened death, serious injury, or sexual violence is provided by the participant.

c. **Participant describes major life event**
This category includes all cases in which the participant describes a major life event, and no further information regarding involvement of actual or threatened death, serious injury, or sexual violence is provided by the participant. Such events may include, but are not limited to, divorce, infidelity, or a parent moving away.

d. **Participant describes event not related to chronic pain, anxiety, nor major life event**
This category includes all cases in which the participant describes an event not related to chronic pain, anxiety, nor a major life event (3a, 3b, or 3c) that does not qualify as Criterion A for PTSD in DSM-5.

4. **Response refers to earlier responses**
This category includes all cases in which the participant refers to an event already reported in one of the preceding 14 items of SLESQ. This might be further elaboration of an event, or any other responses concerning a potentially traumatic event previously

reported. For this category to be applied, it must be clear that the described event is an event reported earlier, and not a similar event not previously reported (e.g., the participant writes that the response is an elaboration of a previously reported event).

5. **Response is not possible to classify**
   This category includes all cases in which the participant provides a response that is not possible to include or discard as Criterion A for PTSD in DSM-5. This might be short responses, or any response not providing sufficient information to determine if the event involved actual or threatened death, serious injury, or sexual violence (e.g., "Surgery" or "My father").

6. **Response regards other aspects of the item or the questionnaire**
   This category includes all cases in which the participant provides a response relevant to the method or theme of item 15 or SLESQ. This might be explanations, corrections, the reason for not answering item 15, or any other response concerning SLESQ.

7. **Response is not related to the item nor the questionnaire**
   This category includes all cases in which the participant provides a response not related to item 15 nor to SLESQ. This might be messages to clinicians, responses referring to other questionnaires, or any other comment irrelevant to SLESQ.

References

1. Goodman, L.A., et al., *Assessing traumatic event exposure: general issues and preliminary findings for the Stressful Life Events Screening Questionnaire.* J Trauma Stress, 1998. **11**(3): p. 521-42.
2. American Psychiatric Association, *Diagnostic and Statistical Manual of Mental Disorders*. 5th ed. 2013, Washington, DC: American Psychiatric Association.

Supplementary File 3

	Total study sample (n = 567		Telephone interviews (n = 55)		Clinical interviews (n = 12)		
	**n**	**%**	**n**	**%**	**n**	**%**	***p***
Gender:							
Male	230	40.6	14	25.5	3	25.0	
Female	337	59.4	41	74.5	9	75.0	.06*
Diagnostic group [1]:							
Primary pain	278	49.1	26	47.3	5	41.7	
Secondary pain	288	50.9	29	52.7	7	58.3	.85*
Secondary pain category [1]:							
Chronic cancer-related pain	3	1.0	0	0	0	0	
Chronic postsurgical or posttraumatic pain	26	9.0	1	3.4	1	14.3	
Chronic secondary musculoskeletal pain	79	27.4	3	10.3	0	0	
Chronic secondary visceral pain	18	6.3	3	10.3	1	14.3	
Chronic neuropathic pain	158	54.9	21	72.4	5	71.4	
Chronic secondary headache or orofacial pain	4	1.4	1	3.4	0	0	.52*
	**M**	**SD**	**M**	**SD**	**M**	**SD**	***p***
Age (17-92/20-78/26-67)	48.14	15.60	48.44	13.99	47.25	14.21	.97*

*Significance not reached

Supplementary Table 1: Groups differences between the total sample and the two subsamples on selected sociodemographics and pain diagnoses.

References

1. IASP Taxonomy working Group. *Classification of Chronic Pain (Second Edition).* 2021 [cited 2022 16. november]; Available from: https://www.iasp-pain.org/publications/free-ebooks/classification-of-chronic-pain-second-edition-revised/?ItemNumber=1673&navItemNumber=677.
